# The Trend of Chronic Diseases Among Older Koreans, 2004-2020: Age-Period-Cohort Analysis

**DOI:** 10.1093/geroni/igaf122.424

**Published:** 2025-12-31

**Authors:** Eun Ha Namkung, Sung Hye Kang

**Affiliations:** Ewha Womans University, Seoul, Republic of Korea; University of California, Los Angeles, Los Angeles, California, United States

## Abstract

This study aimed to examine age, period, and cohort effects contributing to the prevalence of diabetes and hypertension among older Koreans. Additionally, it sought to investigate how sociodemographic characteristics interact with period and cohort effects to influence the disease prevalence. Using the 2004–2020 data from the National Survey of Older Koreans, a nationally representative sample of older adults aged 65 or older, hierarchical age–period–cohort cross-classified random effects models (HAPC-CCREMs) were employed to estimate separate age, period, and cohort components of the recent trends in diabetes and hypertension. Sociodemographic characteristics were tested for their interactions with period and cohort effects. Significant period effects were observed, indicating a steady increase in the likelihood of being diagnosed with diabetes and hypertension over time. Age effects revealed a quadratic trend, with disease risks generally increasing with age, but the rate of increase diminishing at older ages. Cohort effects exhibited an inverted U-shaped pattern, with higher risks observed in the 1930s and early 1940s cohorts compared to earlier and later cohorts. Gender and educational attainment emerged as significant moderators. Women than men born in the early 1930s exhibited higher risks of diabetes and hypertension, whereas individuals with lower educational attainment showed a steadily increasing risk of hypertension over time. The results underscore the complex interplay of age, period, and cohort effects in shaping disease prevalence among older Koreans. Our findings highlight the importance of considering historical context and sociodemographic factors in understanding disease trends and designing targeted interventions to mitigate health disparities.

